# Supplementary material for: Transcription-coupled repair and mismatch repair contribute towards preserving genome integrity at mononucleotide repeat tracts
Source: Nat Commun. 2020 Apr 24;11:1980. doi: 10.1038/s41467-020-15901-w (PMC7181645; doi:10.1038/s41467-020-15901-w)
Supplement: Supplementary file 1 — Supplementary Information [file 41467_2020_15901_MOESM1_ESM.pdf]

Transcription-coupled repair and mismatch repair contribute towards preserving genome integrity at mononucleotide repeat tracts

Georgakopoulos-Soares et al.

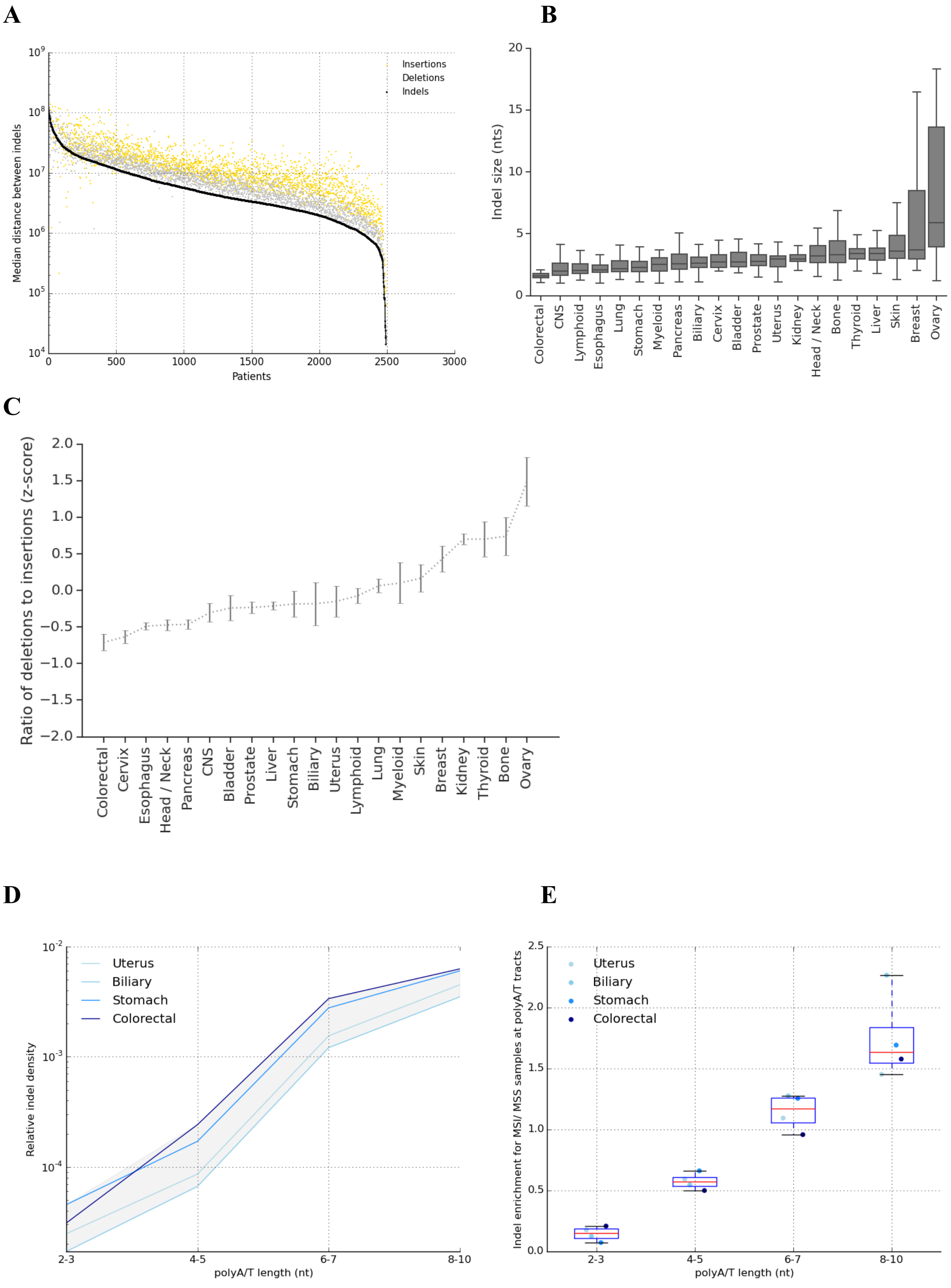
A
B
C
D
E
Insertions
Deletions
Indels
Median distance between indels
Patients
Indel size (nts)
Colorectal
CNS
Lymphoid
Esophagus
Lung
Stomach
Myeloid
Pancreas
Biliary
Cervix
Bladder
Prostate
Uterus
Kidney
Head / Neck
Bone
Thyroid
Liver
Skin
Breast
Ovary
Ratio of deletions to insertions (z-score)
Uterus
Biliary
Stomach
Colorectal
Relative indel density
polyA/T length (nt)
Indel enrichment for MSI/ MSS samples at polyA/T tracts
2-3
4-5
6-7
8-10

Supplementary Figure 1: Indel size distribution patterns by patient and tumour organ. A) Median distance between consecutive indels by patient across tumour types shown in black. Separate analysis of insertion and deletion consecutive distances shown in yellow and grey. Consecutive deletions displayed smaller distance than consecutive insertions (Mann-Whitney U, p-value< 0.001). **B)** Distribution of indel size across patients by tumour type. C) Z-score of the ratio of deletions to insertions for patients in individual cancers compared to the ratio across patients. Standard error is calculated as standard deviation of the ratio in patients within a cancer over the square root of the sample size of the cancer type. Mann-Whitney U tests with Bonferroni correction were performed comparing patients within a cancer type to patients across cancer types and finding significant differences for ovary, kidney, thyroid, bone, breast, esophagus, cervix, CNS and colorectal cancers with p-value<0.001 and lung, skin and head / neck with p-value<0.05. **D)** Relative indel density of indels at polyA/T tracts in relation to the tract length across cancer types (Kruskal-Wallis, p-value<e-07). Here, indel density is defined as the number of indel mutations overlapping polyA/T tracts over the number of bases covered by polyA/T tracts. **E)** Mutational enrichment at MSI over MSS samples for indels at polyA/T tracts in relation to the tract length for endometrial, colorectal, biliary and stomach cancers (Kruskal-Wallis, p-value<0.05).

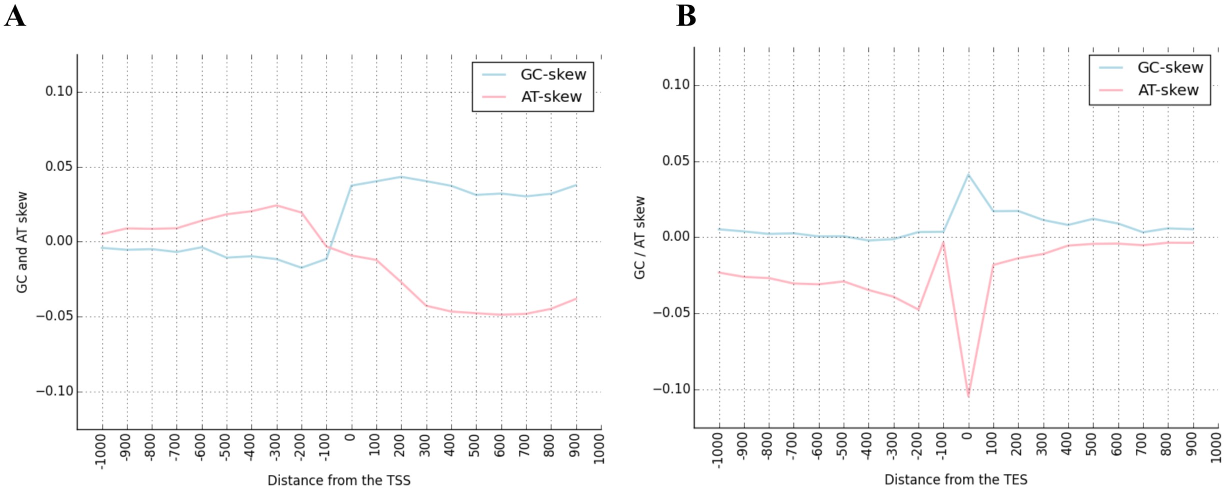

Supplementary Figure 2: GC-skew at the TSS and TES. A) Mean GC-skew and AT-skew around the TSS across genes, **B)** Mean GC-skew and AT-skew around the TES across genes. GC-skew defined as (G-C) / (G+C). AT-skew defined as (A-T) / (A+T).

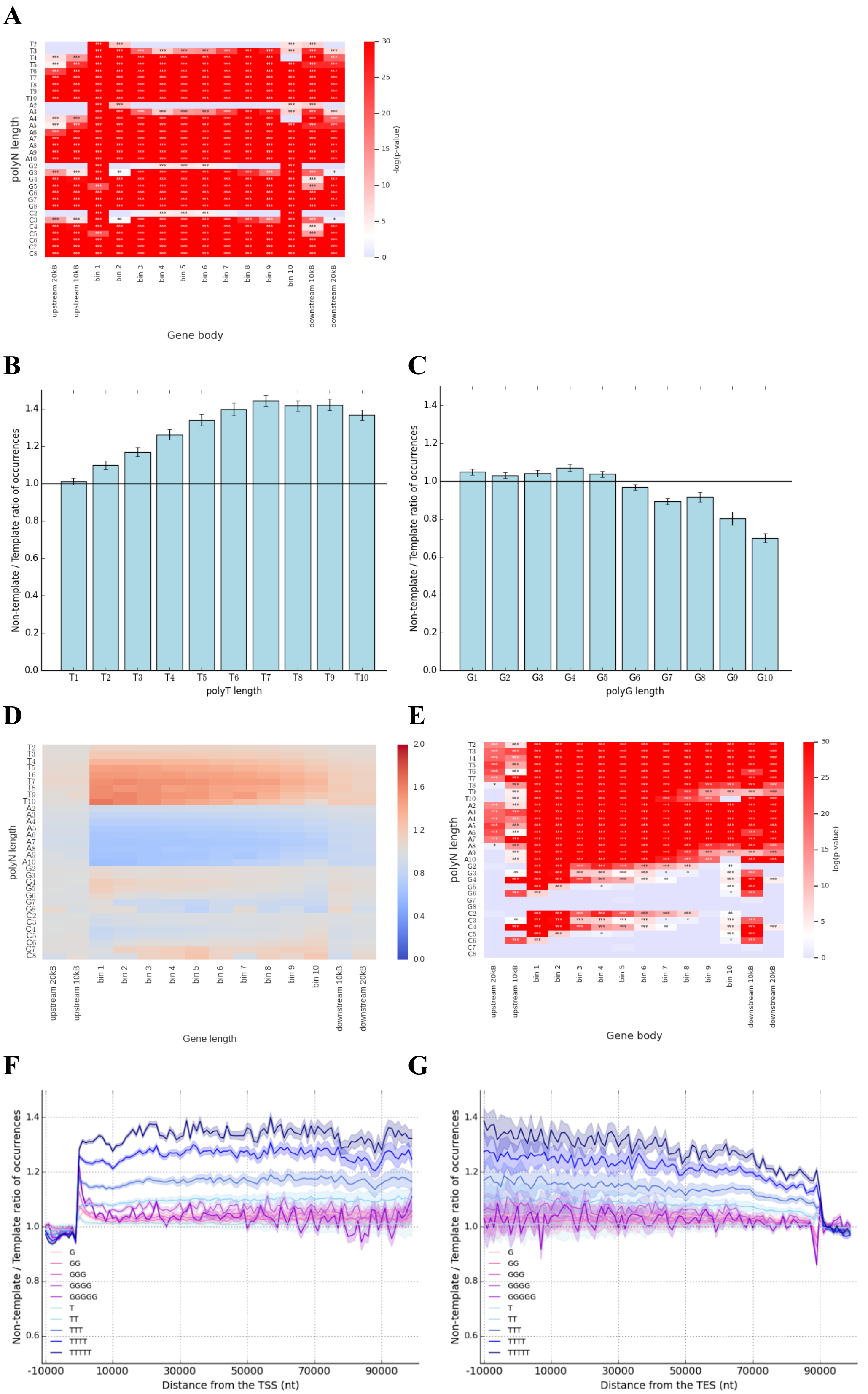
A
polyN length
-log(p-value)
upstream 20kB
upstream 10kB
bin 1
bin 2
bin 3
bin 4
bin 5
bin 6
bin 7
bin 8
bin 9
bin 10
downstream 10kB
downstream 20kB
Gene body
B
Non-template / Template ratio of occurrences
polyT length
T1
T2
T3
T4
T5
T6
T7
T8
T9
T10
C
Non-template / Template ratio of occurrences
polyG length
G1
G2
G3
G4
G5
G6
G7
G8
G9
G10
D
polyN length
Gene length
E
polyN length
-log(p-value)
Gene body
F
Non-template / Template ratio of occurrences
Distance from the TSS (nt)
G
GG
GGG
GGGG
GGGGG
T
TT
TTT
TTTT
TTTTT
G
Non-template / Template ratio of occurrences
Distance from the TES (nt)

Supplementary Figure 3: Transcriptional strand asymmetry of polyN motifs across genic regions. A) Heatmap displaying Mann-Whitney U tests with Bonferroni correction for the density of polyN motifs in each bin versus across the bins. **B)** Non-Template / Template ratio for polyT motifs within the genic region, with error bars representing 1,000-bootstrapping with replacement across genes. **C)** Non-Template / Template ratio for polyG motifs within the genic region, with error bars representing 1,000-bootstrapping with replacement across genes. **D)** Ratio of non-template to template occurrences of polyN motifs across the gene length, red indicating enrichment at non-template and blue indicating enrichment at template strand. **E)** Heatmap displaying Mann-Whitney U tests with Bonferroni correction for the density of polyN motifs at each bin at the template and non-template strands. **F)** Distance from the TSS and non-template /template polyN ratio. **G)** Distance from the TES and non-template /template polyN ratio. Error bars represent standard error from bootstrapping with replacement.

A **B**

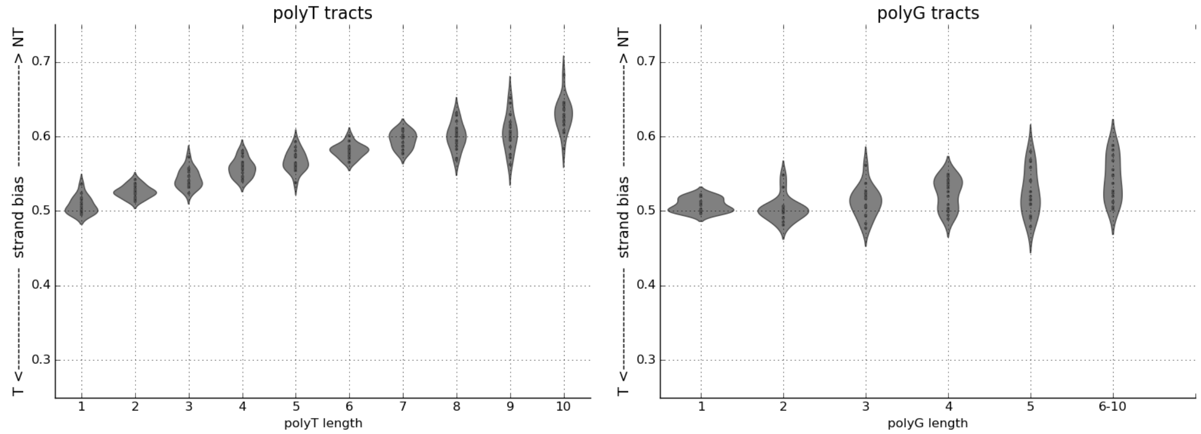

Supplementary Figure 4: Transcriptional strand asymmetry across cancer types and dependence on polyN length for A. polyT tracts, B. polyG tracts. Across cancer types strand asymmetry levels were aggravated for longer polyT tracts at the non-template strand (Kruskal-Wallis H-test with Bonferroni correction p-value<0.001 in all cases). Strand asymmetry levels were dependent on the length of the polyG tracts in lung and liver cancers with a preference towards the non-template strand for longer polyG tracts (Kruskal-Wallis H-test with Bonferroni correction, p-value<0.001 for both) and towards the template strand for longer polyG tracts in pancreatic cancers (Kruskal-Wallis H-test with Bonferroni correction p-value<0.05). For other cancers we could not find an association between strand asymmetry levels and polyG length after multiple testing correction.

A **B**

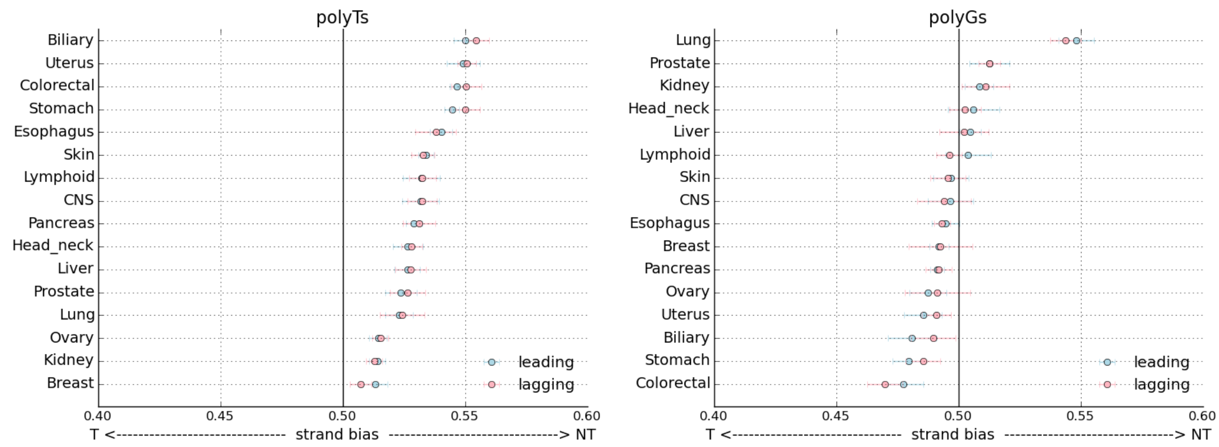

Supplementary Figure 5: Transcriptional strand asymmetry controlling for the direction of the replication fork. Transcriptional-strand asymmetry of indels at polyT and polyG tracts controlling for the effect of replication direction. Transcriptional strand asymmetry at **A.** polyT tracts and **B.** polyG tracts separated by leading and lagging replicative strands, to consider potential confounders due to replication direction. For polyG and polyT tracts the replication direction did not significantly affect the transcriptional strand asymmetry levels (Mann-Whitney U test Bonferroni corrected, p-value>0.05 for all cancer types). X-axis shows transcriptional strand bias (non-template) / (template + non-template), y-axis shows different tissue types. Repli-seq data were derived from MCF-7 cell line. Error bars represent standard deviation from bootstrapping with replacement.

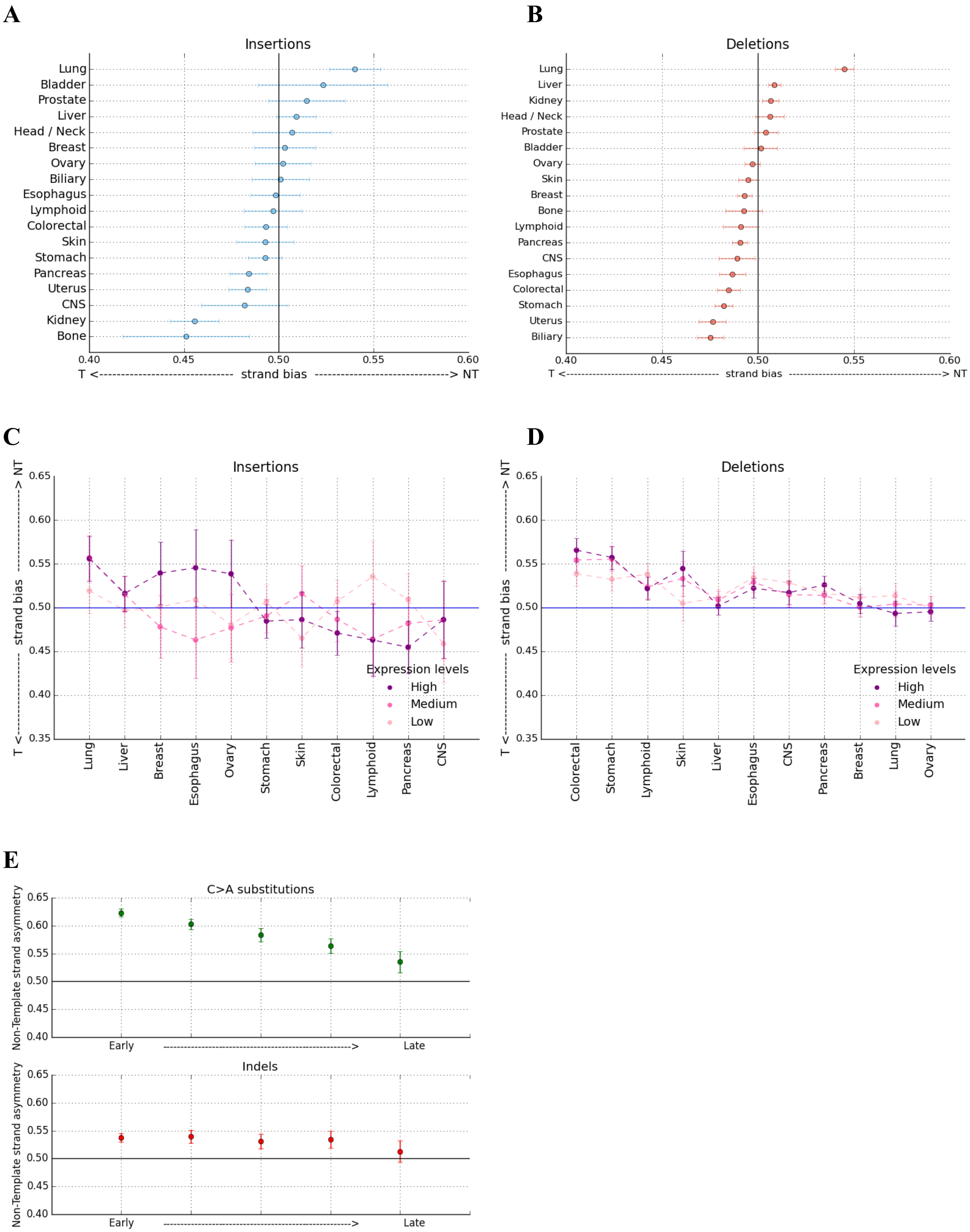
A
Insertions
Lung
Bladder
Prostate
Liver
Head / Neck
Breast
Ovary
Biliary
Esophagus
Lymphoid
Colorectal
Skin
Stomach
Pancreas
Uterus
CNS
Kidney
Bone
0.40
0.45
0.50
0.55
0.60
T <------------------------------ strand bias ------------------------------> NT
B
Deletions
Lung
Liver
Kidney
Head / Neck
Prostate
Bladder
Ovary
Skin
Breast
Bone
Lymphoid
Pancreas
CNS
Esophagus
Colorectal
Stomach
Uterus
Biliary
0.40
0.45
0.50
0.55
0.60
T <------------------------------ strand bias ------------------------------> NT
C
Insertions
T <---------- strand bias ----------> NT
0.65
0.60
0.55
0.50
0.45
0.40
0.35
Lung
Liver
Breast
Esophagus
Ovary
Stomach
Skin
Colorectal
Lymphoid
Pancreas
CNS
Expression levels
High
Medium
Low
D
Deletions
T <---------- strand bias ----------> NT
0.65
0.60
0.55
0.50
0.45
0.40
0.35
Colorectal
Stomach
Lymphoid
Skin
Liver
Esophagus
CNS
Pancreas
Breast
Lung
Ovary
Expression levels
High
Medium
Low
E
C>A substitutions
Non-Template strand asymmetry
0.65
0.60
0.55
0.50
0.45
0.40
Early
-->
Late
Indels
Non-Template strand asymmetry
0.65
0.60
0.55
0.50
0.45
0.40
Early
-->
Late

Supplementary Figure 6: TC-NER and transcriptional strand asymmetry at indels overlapping polyG tracts. A) Transcriptional strand asymmetry at indels overlapping polyG tracts for insertions. **B)** Transcriptional strand asymmetry at indels overlapping polyG tracts for deletions. Error bars represent standard deviation from bootstrapping with replacement. Across cancer types we did not observe a consistent strand asymmetry towards the non-template or the template strand for polyG tracts regarding insertions or deletions (Binomial test with Bonferroni correction, p-value>0.05 for insertions and deletions). (c-d). Transcriptional strand asymmetry at indels overlapping polyG tracts across tumour organs grouped by gene expression levels for cell of origin cell lines. Transcriptional strand asymmetry at: **C)** insertions and **D)** deletions. Mann-Whitney U with Bonferroni correction (p-value>0.05) when comparing low and high expression gene sets across cancer types for insertions and deletions. **E)** Comparing the level of transcriptional strand asymmetry across replication timing domains for substitutions and indels in lung cancer.

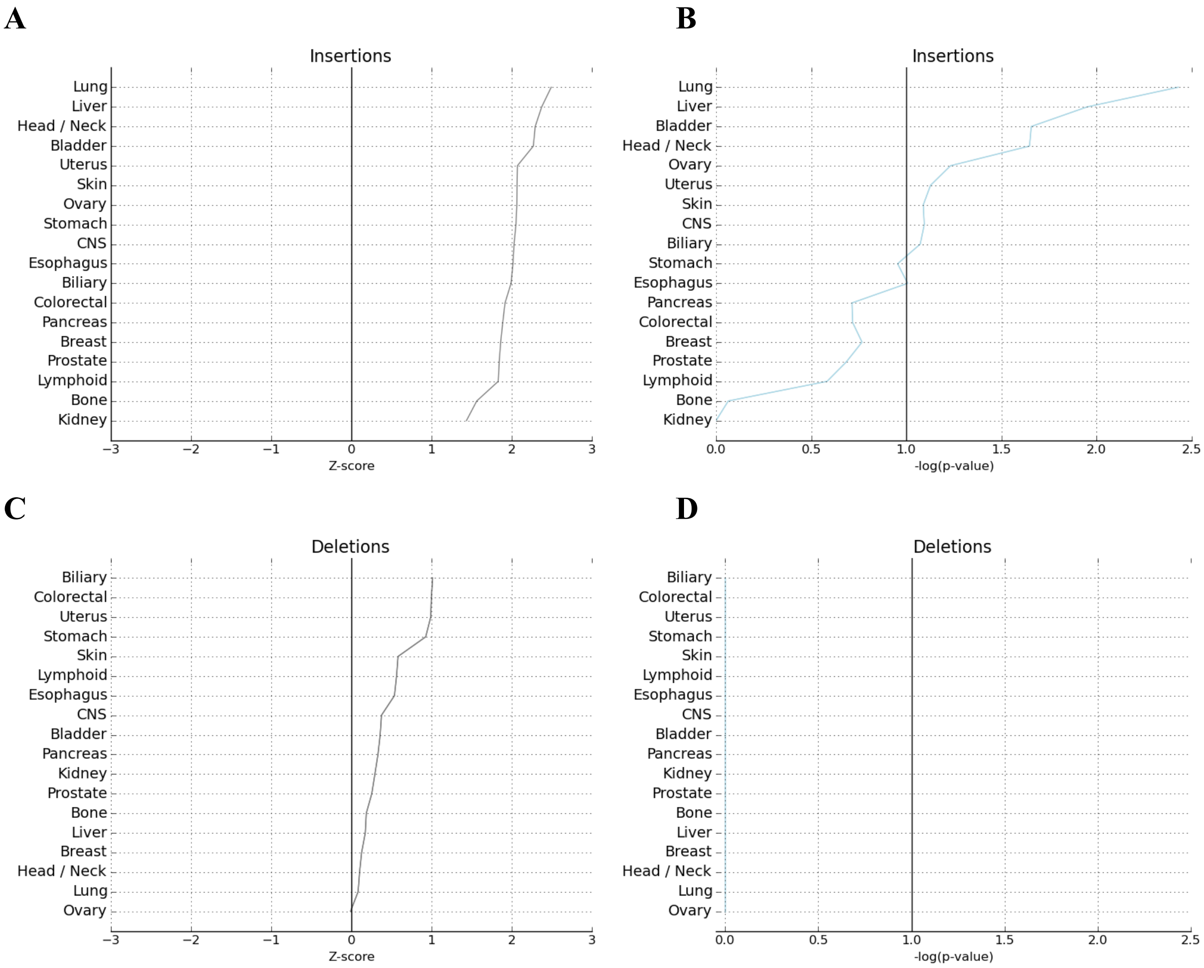

Supplementary Figure 7: Transcriptional strand asymmetry of insertions and deletions for a skin cancer derived from an XPC-deficient patient. A-B. Z-scores and associated p-values from comparing the transcriptional strand asymmetry of the cancer derived from the XPC-deficient patient for insertions overlapping polyT tracts in comparison to the asymmetry at insertions across cancer types. **C-D.** Z-scores and associated p-values from comparing the transcriptional strand asymmetry of the cancer derived from the XPC-deficient patient for deletions overlapping polyT tracts in comparison to the asymmetry at deletions across cancer types. For panels A-D for each cancer type we selected randomly equal number of insertions or deletions overlapping polyT tracts as in the tumour derived from the XPC-deficient patient weighting for the transcriptional asymmetry levels observed at polyT tracts in each cancer type. In this way we controlled for the lower number of insertions or deletions present in the tumour of the XPC-deficient patient. We performed this process 10,000-fold for each cancer from which we calculated the p-values and z-scores from the expected asymmetry for the tumour of the XPC-deficient patient relative to each cancer type.

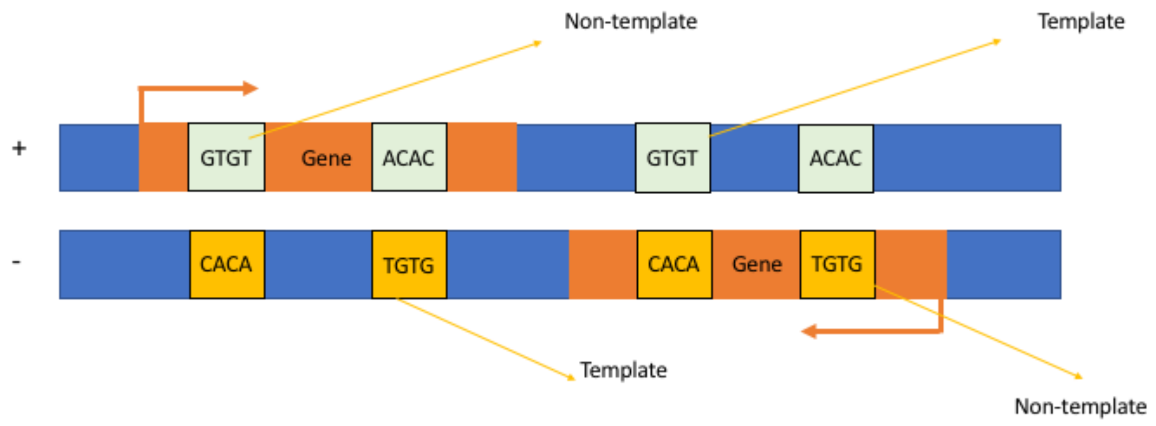

Supplementary Figure 8: Orientation of non-overlapping dinucleotide repeat tracts relative to transcription orientation. Example for the orientation of GTGT tracts relative to the direction of transcription for a gene on the plus strand and a gene on the minus strand, both depicted in dark orange.

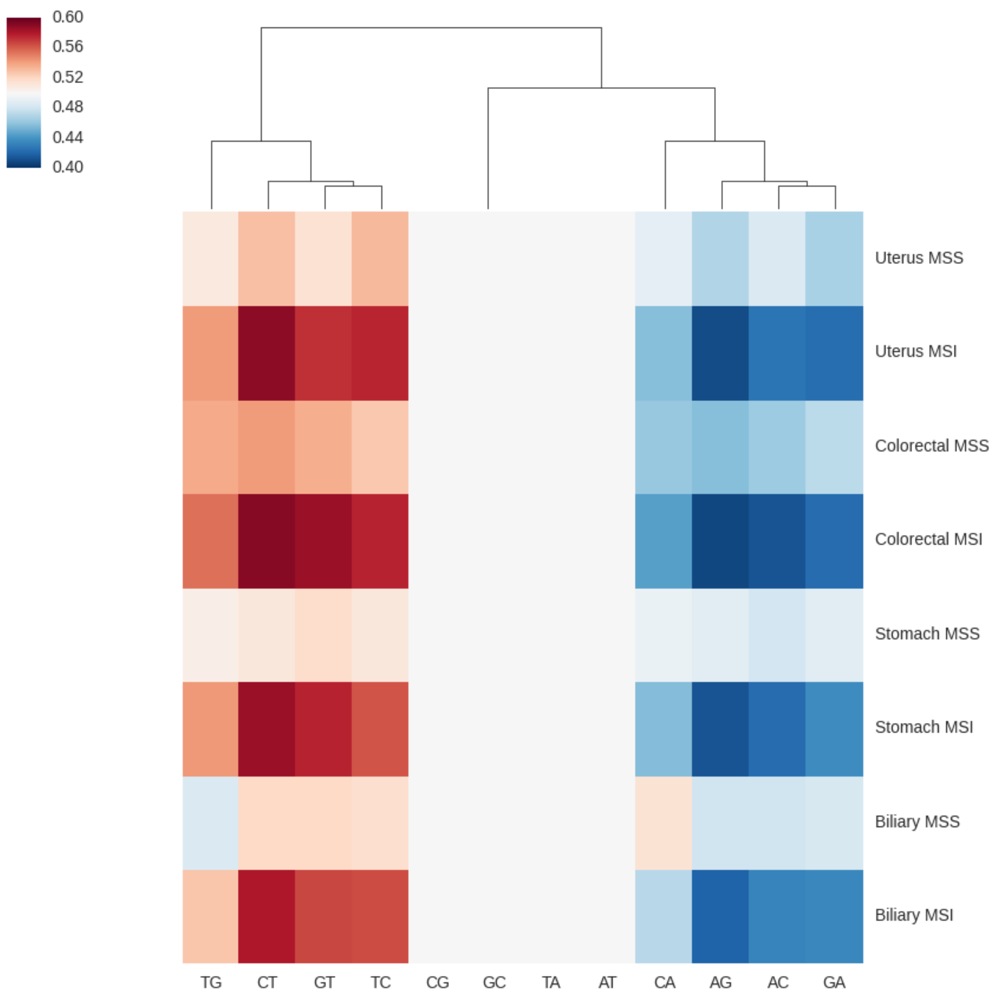

Supplementary Figure 9: Transcriptional strand asymmetries at dinucleotide repeat motifs for MSI and MSS samples of uterus, colorectal, stomach and biliary cancers. The strand asymmetry profile was aggravated for MSI samples (Mann-Whitney U with Bonferroni correction for a number of cancer types and a number of dinucleotide motifs examined, Uterus: p-value<0.001 for GT / AC, CT / AG motifs, Colorectal: p-value<0.05 for GT / AC, CT /AG, TC / GA motifs, Stomach: p-value <0.001 for GT / AC, TC / GA, CT / AG motifs Biliary: p-value <0.05 for TC / GA and CT / AG motifs).

A

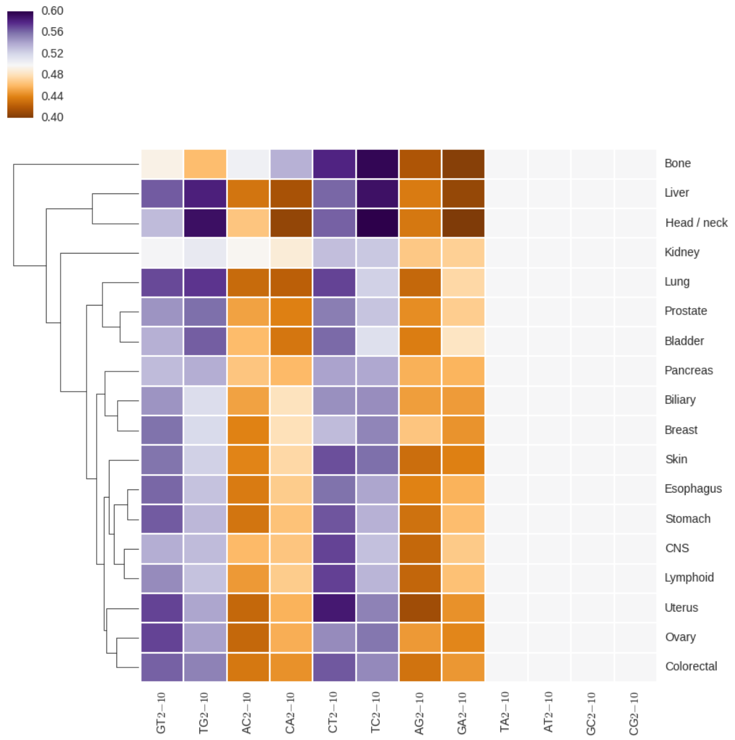
0.60
0.56
0.52
0.48
0.44
0.40
Bone
Liver
Head / neck
Kidney
Lung
Prostate
Bladder
Pancreas
Biliary
Breast
Skin
Esophagus
Stomach
CNS
Lymphoid
Uterus
Ovary
Colorectal
GT2−10
TG2−10
AC2−10
CA2−10
CT2−10
TC2−10
AG2−10
GA2−10
TA2−10
AT2−10
GC2−10
CG2−10

B

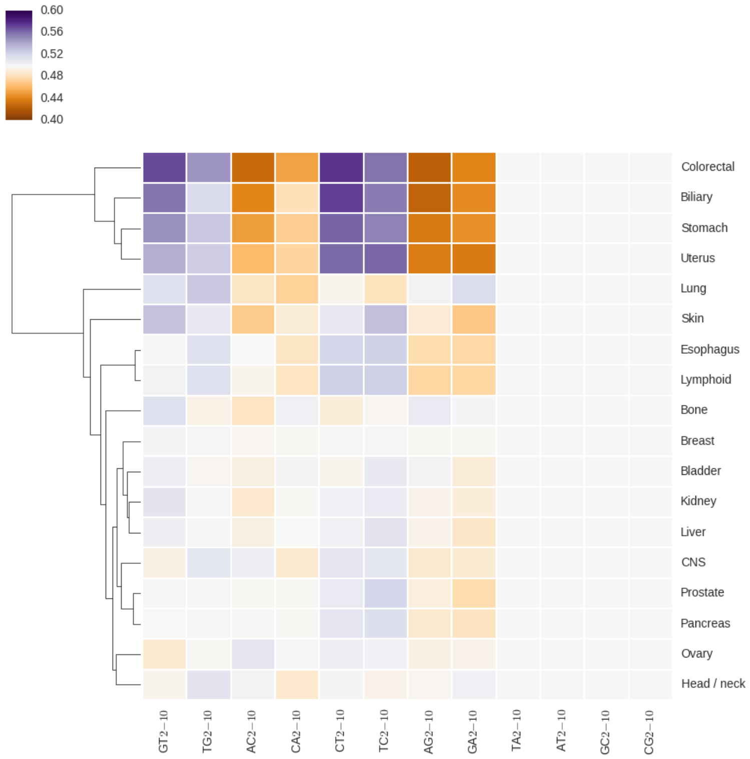
0.60
0.56
0.52
0.48
0.44
0.40
Colorectal
Biliary
Stomach
Uterus
Lung
Skin
Esophagus
Lymphoid
Bone
Breast
Bladder
Kidney
Liver
CNS
Prostate
Pancreas
Ovary
Head / neck
GT2−10
TG2−10
AC2−10
CA2−10
CT2−10
TC2−10
AG2−10
GA2−10
TA2−10
AT2−10
GC2−10
CG2−10

Supplementary Figure 10: Transcriptional strand asymmetries across dinucleotide repeat motifs for A) insertions, and B) deletions across cancer types. Wilcoxon signed-rank with Bonferroni correction indicating significant differences in the strand asymmetry levels at insertions, p-value<0.05 for GT / AC and TG / CA dinucleotides and p-value < 0.001 for CT / AG and TC / GA. Wilcoxon signed-rank with Bonferroni correction indicating significant differences in the strand asymmetry levels at deletions, p-value<0.05 for CT / AG, TC / GA and TG / CA dinucleotides.

Supplementary Table 1: Number of patients, insertions and deletions per tumour organ.

Tumour organ	Patients	Deletions	Insertions
Bladder	23	11,101	5,571
Biliary	34	119,952	35,024
Pancreas	313	93,936	91,392
Head / Neck	56	23,756	14,602
Liver	314	150,392	78,977
Ovary	110	59,917	27,903
Prostate	199	36,017	22,512
Colorectal	52	208,761	132,204
Myeloid	38	1,177	609
Stomach	68	253,355	62,045
Cervix	20	3,854	3,434
Uterus	44	119,848	78,578
CNS	287	29,362	19,497
Lymphoid	197	61,209	43,592
Skin	107	79,358	27,657
Kidney	186	104,359	29,518
Breast	211	70,333	23,088
Esophagus	97	89,741	63,642
Thyroid	48	3,101	1,045
Bone	89	14,256	4,527
Lung	82	89,842	34,210

Supplementary Table 2: Cell of origin RNA-seq datasets from [36] that were used to calculate the transcription strand asymmetry levels of cancer organs for genes of different expression levels.

Cancer	Cell type
Breast	MCF-7 cell line
Colorectal	Sigmoid colon primary cells
Lung	IMR-90 cell line
Pancreas	Pancreatic primary cells
Liver	HepG2 cell line
Esophagus	Esophageal primary cells
Ovarian	Ovary primary cells
CNS	Female fetal brain cells
Lymphoid	K562 cell line
Skin	Foreskin fibroblasts
Stomach	Gastric primary cells
