# Supplementary material for: Transcription-coupled repair and mismatch repair contribute towards preserving genome integrity at mononucleotide repeat tracts
Source: Nat Commun. 2020 Apr 24;11:1980. doi: 10.1038/s41467-020-15901-w (PMC7181645; doi:10.1038/s41467-020-15901-w)
Supplement: Supplementary file 3 — Reporting Summary [file 41467_2020_15901_MOESM3_ESM.pdf]

nature research

Corresponding author(s): Dr. Martin Hemberg and Dr. Serena Nik-Zainal

Last updated by author(s): Mar 11, 2020

Reporting Summary

Nature Research wishes to improve the reproducibility of the work that we publish. This form provides structure for consistency and transparency in reporting. For further information on Nature Research policies, see Authors & Referees and the Editorial Policy Checklist.

Statistics

For all statistical analyses, confirm that the following items are present in the figure legend, table legend, main text, or Methods section.

n/a	Confirmed	
☐	☒	The exact sample size (*n*) for each experimental group/condition, given as a discrete number and unit of measurement
☐	☒	A statement on whether measurements were taken from distinct samples or whether the same sample was measured repeatedly
☐	☒	The statistical test(s) used AND whether they are one- or two-sided<br>*Only common tests should be described solely by name; describe more complex techniques in the Methods section.*
☐	☒	A description of all covariates tested
☐	☒	A description of any assumptions or corrections, such as tests of normality and adjustment for multiple comparisons
☐	☒	A full description of the statistical parameters including central tendency (e.g. means) or other basic estimates (e.g. regression coefficient) AND variation (e.g. standard deviation) or associated estimates of uncertainty (e.g. confidence intervals)
☐	☒	For null hypothesis testing, the test statistic (e.g. *F*, *t*, *r*) with confidence intervals, effect sizes, degrees of freedom and *P* value noted<br>*Give P values as exact values whenever suitable.*
☐	☒	For Bayesian analysis, information on the choice of priors and Markov chain Monte Carlo settings
☐	☒	For hierarchical and complex designs, identification of the appropriate level for tests and full reporting of outcomes
☐	☒	Estimates of effect sizes (e.g. Cohen's *d*, Pearson's *r*), indicating how they were calculated

Our web collection on statistics for biologists contains articles on many of the points above.

Software and code

Policy information about availability of computer code

Data collection	Mutation data tables and code can be found here: https://data.mendeley.com/datasets/kdywxnn729 as well as in https://github.com/IliasGeoSo/Transcriptional_strand_asymmetry_Indels
Data analysis	Python 2.7.15 was used throughout the analysis. BEDTools utilities v2.21.0 was used to manipulate genomic files. The python packages seaborn version 0.9.0, numpy version 1.16.2, scipy version 1.2.1, matplotlib version 1.4.2, json version 2.0.9, pandas version 0.22.0 were used for analysis and plotting. JBrowse Genome Browser was used to inspect mapping quality. bsub LSF was used to submit parallel jobs.

For manuscripts utilizing custom algorithms or software that are central to the research but not yet described in published literature, software must be made available to editors/reviewers. We strongly encourage code deposition in a community repository (e.g. GitHub). See the Nature Research guidelines for submitting code & software for further information.

Data

Policy information about availability of data

All manuscripts must include a data availability statement. This statement should provide the following information, where applicable:

- Accession codes, unique identifiers, or web links for publicly available datasets
- A list of figures that have associated raw data
- A description of any restrictions on data availability

Mutation data tables and code can be found here: https://data.mendeley.com/datasets/kdywxnn729/3/
Primary mutation data were obtained from ICGC under the project PanCancer Analysis of Whole Genomes (PCAWG). A cutaneous malignancy derived from a patient with an autosomal recessive DNA repair defect called Xeroderma Pigmentosum (XP) mutation was obtained from Momen et al. 2019). Indel mutational profiles of non-cancerous human cells exposed to various polycyclic aromatic hydrocarbons (PAHs) including benzo[a]pyrene [0.39 µM and 2 µM] and benzo[a]pyrene diol epoxide [0.125 µM] were derived from ftp://ftp.sanger.ac.uk/pub/cancer/Zou_et_al_2017 and experimentally-generated mutation patterns

from CRISPR-Cas9 knockouts of a human cancer cell line for MSH6 were derived from https://data.mendeley.com/datasets/m7r4msjb4c/2 .

Field-specific reporting

Please select the one below that is the best fit for your research. If you are not sure, read the appropriate sections before making your selection.

☒ Life sciences ☐ Behavioural & social sciences ☐ Ecological, evolutionary & environmental sciences

For a reference copy of the document with all sections, see nature.com/documents/nr-reporting-summary-flat.pdf

Life sciences study design

All studies must disclose on these points even when the disclosure is negative.

Sample size	Sample size of each tumour type was the number of patients for that tumour type (Supplementary Table 1).
Data exclusions	Myeloid, cervix and thyroid cancers were excluded due to low numbers of total indels for transcriptional strand asymmetry analysis (Supplementary Table 1).
Replication	The analysis of transcriptional strand asymmetry was performed using bootstrapping of randomly selected equal number of genes. A second approach used was by simulating indels and calculating the asymmetry. The two approaches showed comparable results. The results remained the same when also controlling for the effect of replication orientation.
Randomization	Patients were aggregated into groups by tissue of tumour, resulting in 21 groups. Analyses were performed in each tissue. Also, cancer patients were separated by repair enzyme deficiency in MSI and MSS samples for uterus, colorectal, stomach and biliary cancers.
Blinding	Blinding was not applicable in our study.

Reporting for specific materials, systems and methods

We require information from authors about some types of materials, experimental systems and methods used in many studies. Here, indicate whether each material, system or method listed is relevant to your study. If you are not sure if a list item applies to your research, read the appropriate section before selecting a response.

Materials & experimental systems

n/a	Involved in the study
☒	☐ Antibodies
☒	☐ Eukaryotic cell lines
☒	☐ Palaeontology
☒	☐ Animals and other organisms
☒	☐ Human research participants
☒	☐ Clinical data

Methods

n/a	Involved in the study
☒	☐ ChIP-seq
☒	☐ Flow cytometry
☒	☐ MRI-based neuroimaging
